# SMR peptide antagonizes *Staphylococcus aureus* biofilm formation

**DOI:** 10.1128/spectrum.02583-23

**Published:** 2024-01-03

**Authors:** Ming-Bo Huang, Dara Brena, Jennifer Y. Wu, Martin Shelton, Vincent C. Bond

**Affiliations:** 1 Department of Microbiology, Biochemistry, and Immunology, Morehouse School of Medicine, Atlanta, Georgia, USA; 2 Columbia University School of International and Public Affairs, Columbia University, New York, New York, USA; 3 NanoString Technologies, Inc, Seattle, Washington, USA; The Hebrew University of Jerusalem, Rehovot, Israel

**Keywords:** bacterial biofilm, *Staphylococcus aureus*, SMR peptide, heat shock protein, DnaK

## Abstract

The emergence and international dissemination of multi-drug resistant *Staphylococcus aureus* (*S. aureus*) strains challenge current antibiotic-based therapies, representing an urgent threat to public health worldwide. In the U.S. alone, *S*. *aureus* infections are responsible for 11,000 deaths and 500,000 hospitalizations annually. Biofilm formation is a major contributor to antibiotic tolerance and resistance-induced delays in empirical therapy with increased infection severity, frequency, treatment failure, and mortality. Developing novel treatment strategies to prevent and disrupt biofilm formation is imperative. In this article, we test the Secretion Modification Region (SMR) peptides for inhibitory effects on resistant *S. aureus* biofilm-forming capacity by targeting the molecular chaperone DnaK. The dose effect of SMR peptides on biofilm formation was assessed using microtiter plate methods and confocal microscopy. Interaction between the antagonist and DnaK was determined by immune precipitation with anti-Flag M2 Affinity and Western blot analysis. Increasing SMR peptide concentrations exhibited increasing blockade of *S. aureus* biofilm formation with significant inhibition found at 18 µM, 36 µM, and 72 µM. This work supports the potential therapeutic benefit of SMR peptides in reducing biofilm viability and could improve the susceptibility to antimicrobial agents.

**IMPORTANCE:**

The development of anti-biofilm agents is critical to restoring bacterial sensitivity, directly combating the evolution of resistance, and overall reducing the clinical burden related to pervasive biofilm-mediated infections. Thus, in this study, the SMR peptide, a novel small molecule derived from the HIV Nef protein, was preliminarily explored for anti-biofilm properties. The SMR peptide was shown to effectively target the molecular chaperone DnaK and inhibit biofilm formation in a dose-dependent manner. These results support further investigation into the mechanism of SMR peptide-mediated biofilm formation and inhibition to benefit rational drug design and the identification of therapeutic targets.

## INTRODUCTION

### Global clinical burden of *Staphylococcus aureus*

*Staphylococcus aureus* (*S. aureus*) is a ubiquitous gram-positive bacterium responsible for a plethora of community and nosocomial infections ranging from mild skin and soft tissue infections to life-threatening conditions, including pneumonia, meningitis, osteomyelitis, endocarditis, bacteremia, and toxic shock syndrome (1–3). The high abundance of *S. aureus* within the environment as well as within the flora of humans and animal species contribute to a large reservoir of virulence factors and antibiotic resistance tactics (4). Over the years, *S. aureus* has developed resistance to multiple antibiotics and has been designated as an ESKAPE (*Enterococcus faecium, Staphylococcus aureus, Klebsiella pneumoniae, Acinetobacter baumannii, Pseudomonas aeruginosa,* and *Enterobacter* species) pathogen (5, 6). According to the CDC’s Antibiotic Resistance Threats Report, *S. aureus* is continuing to readily disseminate dangerous antimicrobial resistance genes through mobile genetic elements (7, 8). The global spread of types and subtypes of methicillin-resistant *S. aureus* (MRSA) has led to an expanding public health crisis (9). Patients with MRSA frequently have chronic infections with a high risk for bacterial seeding to critical secondary sites resulting in severe acute infections and death (9). In a 2019 global mortality study analyzing 33 bacterial pathogens, *S. aureus* ranked within the top five causes of death (10).

### *S. aureus* biofilm formation promotes antimicrobial tolerance and resistance

A large degree of the clinical burden due to *S. aureus* can be attributed, as Reffuveille et al. refer, to the “superstructure” of biofilms (11). Approximately 80% of the *S. aureus* nosocomial infections are biofilm associated (11). *S. aureus* biofilms inherently exhibit recalcitrance to treatment, thereby increasing the severity, recurrence, chronicity, and mortality of infections. *S. aureus* biofilms perpetuate both antimicrobial tolerance and resistance by two primary mechanisms as follows: (i) the biofilm acts as a protective barrier against physical stress, antibiotics, and host immune response. Bacteria living in a biofilm can exhibit a 10- to 1,000-fold increase in antibiotic tolerance compared to similar bacteria living in a planktonic state (12). (ii) The biofilm serves as a platform for complex microbial communal networking and antimicrobial resistance transmission. It is important to distinguish between a biofilm’s capacity for antibiotic tolerance vs resistance. Antibiotic tolerance is a temporary state of reduced susceptibility to antibiotics, often associated with biofilms, whereas antibiotic resistance involves genetic changes that enable bacteria to grow and divide in the presence of antibiotics (13).

During biofilm formation, bacteria produce and surround themselves in an extracellular polymeric substance (EPS) consisting of polysaccharides, lipids, nucleic acids, and protein (14). EPS establishes the functional and structural integrity of a biofilm, acting as a key mediator for physiochemical interactions. Antimicrobial tolerance at the biofilm surface is due in part to this EPS matrix that limits antibiotic diffusion. Furthermore, any antibiotics that penetrate the biofilm are unlikely to maintain functionality. Proteolytic enzymes, metabolic byproducts, and other environmental factors of the biofilm compromise antibiotic structure and action (15). For example, low oxygen levels reduce the bactericidal effects of antibiotics tobramycin and ciprofloxacin, whereas pH changes can negatively impact aminoglycoside action. Persister or dormant bacterial subpopulations within the biofilm enter a “spore-like” state and are tolerant to extreme conditions, such as chemical treatment and antibiotic activity that act by targeting cell division. The survival of these persister cells is not due to any genetic changes; upon release from the biofilm, they begin dividing again and return to their pre-persister susceptibility profiles (16–31). Antimicrobial tolerance and resistance within the biofilm are attributed to the heterogeneity of the bacterial populations and the proximity of the bacterial cells that facilitate a highly active transmission of resistant genetic elements. Conjugation and horizontal gene transfer are the predominant modes that reinforce pervasive phenotypic traits throughout the bacterial populations. For example, efflux pumps are readily disseminated through horizontal gene transfer. Despite the health burden due to *S. aureus* biofilms, many antibiotic assays for susceptibility and resistance are based on planktonic cells, and there are few therapies that prevent and/or disrupt biofilm formation (32, 33).

### Clinical relevance of antimicrobial peptides (AMPs)

AMPs are considered a promising alternative to conventional antibiotics (34). AMPs exhibit broad-spectrum activity and a robust bactericidal effect on multi-drug resistant (MDR) microorganisms (34). However, the use of AMPs requires a comprehensive understanding of the complex interactions between peptides, bacterial resistance mechanisms, and host factors (34, 35). Notable disadvantages that limit the clinical application of AMPs include difficulties in maintaining effective levels (e.g., proteolytic degradation, fast clearance by the kidney and liver, etc.) and toxicity (34). Additionally, AMPs are subject to bacterial resistance development (36). For example, bacteria can alter their membrane composition, charge, or surface molecules to prevent effective targeting by AMPs (36). Synergistic approaches that combine AMPs with therapeutic agents such as antibiotics or other AMPs may offer a more effective strategy to prevent resistance development and enhance antimicrobial activity (36). Continued research efforts into AMPs to improve efficacy and bioavailability, reduce toxicity, and limit the acquisition of resistance will aid in combatting the antimicrobial-resistance health crisis (34).

### Rationale of SMR peptide targeting of DnaK for biofilm disruption

DnaK is a molecular chaperone within the heat shock protein 70 family. DnaK acts by binding nascent polypeptide chains and assists in protein assembly, refolding, maintenance, and degradation (37). Although the mechanism is unclear, DnaK has been identified as important to biofilm formation and is predicted to be critical for the appropriate folding of biofilm scaffolding proteins. For example, in *Escherichia coli*, DnaK contributed to amyloid curli expression. In turn, DnaK represents a potential therapeutic target for inhibition to disrupt and prevent the formation of biofilms (38, 39).

**Fig 1:**
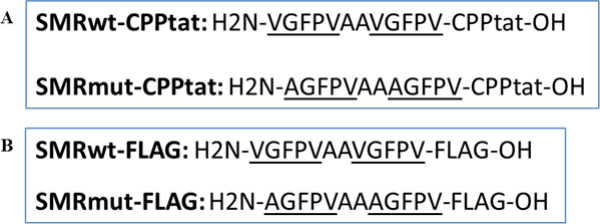
Design and development of the SMR peptides. (**A**) The difference between SMRwt-CPPtat and SMRmut-CPPtat is that SMRwt-CPPtat contains a valine, whereas SMRmut-CPPtat contains an alanine at the first position from the N-terminus. CPPtat stands for Cell-Penetrating Peptide Tat and comprises arginine and lysine residues. CPPtat is a potent transduction agent that can penetrate cell membranes and efficiently transport cargo into cells. (**B**) The SMRwt-FLAG peptide has an N-terminus histidine and a C-terminus hydroxylamine, followed by the FLAG epitope tag. The SMRmut-FLAG peptide is the same as the wild-type peptide, but with a point mutation at the first amino acid from the N-terminus, changing the valine to an alanine.

Our lab group has designed and developed a series of SMR peptides to target Mortalin, a heat shock protein with a high degree of homology to DnaK (37, 40, 41). In this study, we aim to explore the effects on biofilms from targeting DnaK with the SMR peptides. The SMRwt and SMRmut peptide sequences are depicted in Fig. 1. SMRmut, a negative control, has an alanine substitution that inactivates the function of the antagonist peptide. Modification of the SMR peptide with a 13-mer cell-penetrating peptide (CPP) derived from the HIV-1 Tat protein in C-terminus enables penetration through bacterial cell membranes. The SMR-FLAG is the same as the SMR-CPPtat with a FLAG sequence replacing the CPP (42, 43).

In our previous studies, we have found multiple novel effects of the SMR peptides through targeting mortalin in HIV/AIDS and breast cancer (28). Similar to DnaK, mortalin is involved in the translocation and folding of proteins to regulate many processes, including cell growth, apoptosis, and autophagy. Mortalin also interacts with proteins, such as Nef, to elicit the secretion of extracellular vesicles (EV) that mediate intercellular communication and environmental response coordination. Both antibody inhibition and miRNA knockdown experiments confirmed that blocking mortalin blocks Nef-EV secretion in Nef-transfected cells, as well as disrupts mortalin/Nef interactions occurring through the SMR domain (28). Our recent research demonstrated that SMR peptide treatment in breast cancer cells blocked EV secretion, induced cell cycle arrest at the G2/M phase, and reduced cellular proliferation (40, 41). We hypothesized that SMR peptide treatment of resistant *S. aureus* will interact with DnaK, like mortalin, and block both the folding of scaffolding proteins and EV release, to compromise the structural integrity of the biofilm and the EV-mediated intercellular networking, respectively. We addressed this hypothesis by microtiter plate (MtP) assays, immunoprecipitation assays, and confocal microscopy. This study offers insights into the development of novel treatment strategies to mitigate the impact of this versatile and resilient pathogen.

## RESULTS

### SMRwt peptide interacts with DnaK in *E*. *coli* and *S. aureus* strains

**Fig 2:**
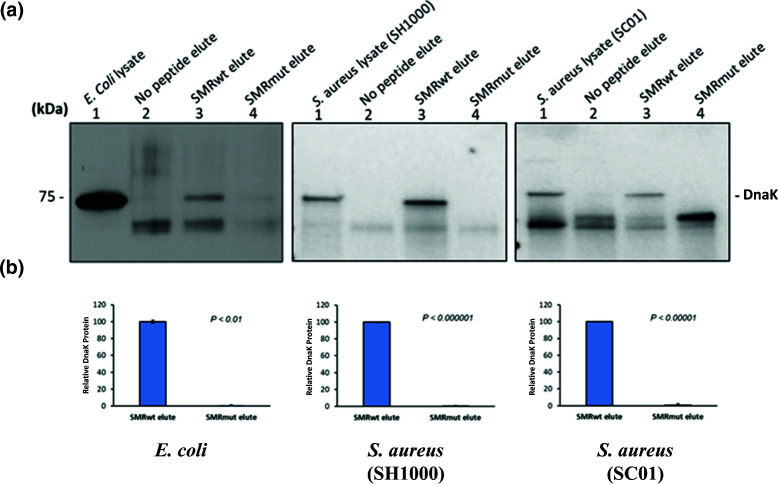
SMR peptides interact with target DnaK in *E. coli* and *S. aureus* strains. (**A**) Western blot analysis of the DnaK antibody depicted the interaction between the SMRwt and DnaK following an immunoprecipitation assay. *E. coli* (Invitrogen MAX efficiency Stbl2 competent), *S. aureus* (SH1000), and *S. aureus* (SC01) were grown in media without antibiotics. The cells were lysed and the resultant lysate was screened by an Anti-Flag M2 affinity under three conditions as follows: SMRwt, SMRmut, and the absence of both peptides. (**B**) Bar graphs depict the SMRwt and SMRmut elute band percentage intensity. Results are presented as means ± standard deviations (*n* = 3).

The interaction between the SMRwt peptide and DnaK was studied using an immunoprecipitation assay. In brief, *E. coli* (Invitrogen MAX Efficiency Stbl2 Competent cells), *S. aureus* (SH1000), and *S. aureus* (SC01) cell lysates were screened with an anti-flag M2 affinity gel under three different conditions as follows: SMRwt vs SMRmut vs no peptide. The bound proteins were eluted and subsequently separated using SDS-PAGE. The Western blot analysis with the DnaK antibody revealed a SMRwt band, which was absent under the SMRmut and no peptide conditions (Fig. 2A). A significantly higher elution percentage was observed for SMRwt compared to SMRmut (Fig. 2B). Specifically, the *P*-values were as follows: *E. coli* (Invitrogen MAX Efficiency Stbl2 Competent cells) *P*-value < 0.01, *S. aureus* (SH1000) *P*-value < 0.000001, and *S. aureus* (SC01) *P*-value < 0.00001. This indicated that the SMRwt peptide was interacting with DnaK in a manner that increased its retention in the affinity gel that was not seen for SMRmut.

### SMRwt peptide significantly inhibits *S. aureus* biofilm formation

**Fig 3:**
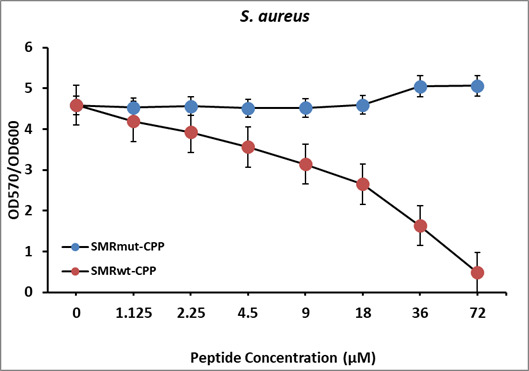
SMR peptides inhibit *S. aureus* biofilm formation in a dose-dependent manner. *S. aureus* (SC01) was grown at 37°C to the stationary phase. The cultures were diluted 100-fold in fresh medium and seeded into a 96-well microtiter plate with either SMRwt or SMRmut at the indicated concentrations. After 24 hours, biofilm formation was measured using the crystal violet staining method (OD570). The data were normalized for growth (OD600). Error bars represent the mean ± SD of two independent experiments. Significant differences relative to the negative control were noted at SMR peptide concentrations of 36 µM with a *P*-value < 0.022 and 72 µM with a *P*-value < 0.015.

**Fig 4:**
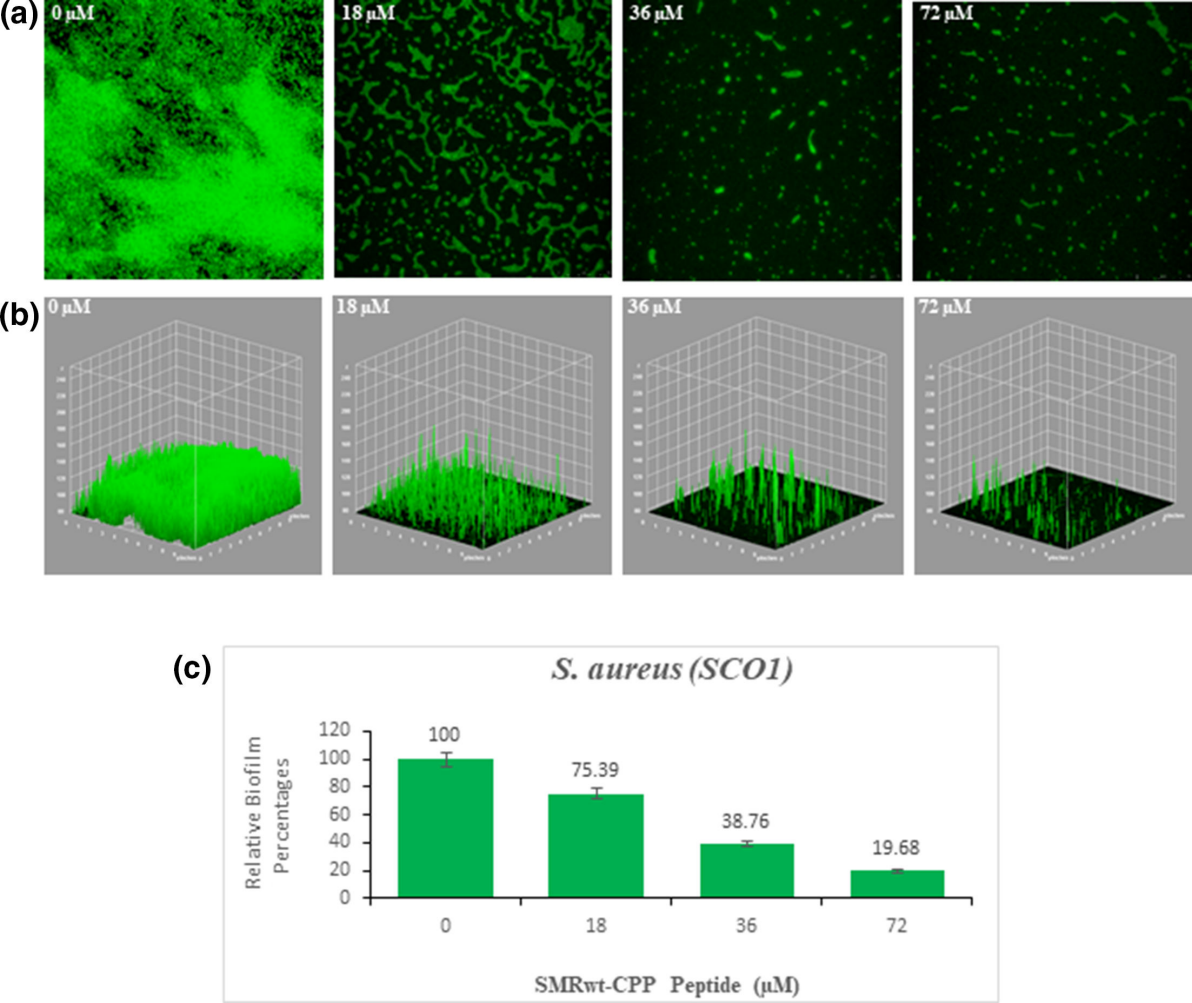
Confocal microscopy and three-dimensional (3D) views of *S. aureus* biofilms reveal effective SMR peptide antagonism. *S. aureus* (SC01) was grown at 37°C to the stationary phase. The cultures were diluted 100-fold in fresh media and seeded onto a MatTek glass bottom plate. After 24 hours, the biofilms were stained with SYTO9, observed by Leica confocal microscopy, and analyzed with ImageJ. (**A**) Confocal images, (**B**) 3D images, and (**C**) bar graphs depict the inhibitory effect of SMR peptides on *S. aureus* biofilm formation. Error bars represent the mean ± SD of three independent experiments. Significant differences relative to the negative control were noted at SMR peptide concentrations of 18 µM with a *P*-value < 0.05 as well as 36 µM and 72 µM with a *P*-value < 0.005.

The SMRwt peptide effectively blocked biofilm formation of *S. aureus* (SC01) as shown through MtP methods and confocal microscopy (Fig. 3 and 4). For the MtP assays, *S. aureus* (SC01) was seeded with either SMRwt or SMRmut, and biofilm formation was measured using the crystal violet staining method (OD570). The data were normalized for growth (OD600). A clear inhibitory effect was identified with increasing SMRwt concentrations contributing to significantly decreased biofilm formation (Fig. 3). Significant differences relative to the negative control were noted at SMR peptide concentrations of 36 µM with a *P*-value < 0.022 and 72 µM with a *P*-value < 0.015. Three-dimensional confocal microscopy findings reinforced this premise of SMR peptide-mediated biofilm formation (Fig. 4B). The dose effect observed through confocal microscopy aligned strongly with the crystal violet staining findings. Specifically, SMR dosages 18 µM, 36 µM, and 72 µM resulted in significantly decreased biofilm formation of 75.39%, 38.76%, and 19.68%, respectively (Fig. 4C).

## DISCUSSION

*S. aureus* biofilms facilitate life-threatening infections recalcitrant to antibiotic therapy, contributing to a substantial burden on healthcare systems worldwide (44–47). The intrinsic and acquired biofilm-specific mechanisms for tolerance and resistance against antimicrobial agents, host immune response, and environmental stressors reinforce persistent and severe nosocomial infections (45). Despite these clinical threats of *S. aureus* biofilm-associated infections, currently, there are no anti-biofilm treatments available (48). Thus, there have been intensive research efforts on the discovery and development of agents to prevent and/or disrupt biofilms. DnaK has been identified as a promising target with its chemical inhibition resulting in effective biofilm prevention (38, 49). Elimination of DnaK functionality through either gene deletion or direct inhibition has been found to reduce adherence, the production of amyloid curli, as well as the robustness of the biofilm in both *E. coli* and *S. aureus* (38). Furthermore, in a murine model, DnaK blockade diminished *S. aureus* resistance to unfavorable environmental factors and overall survivability (50, 51).

**Fig 5:**
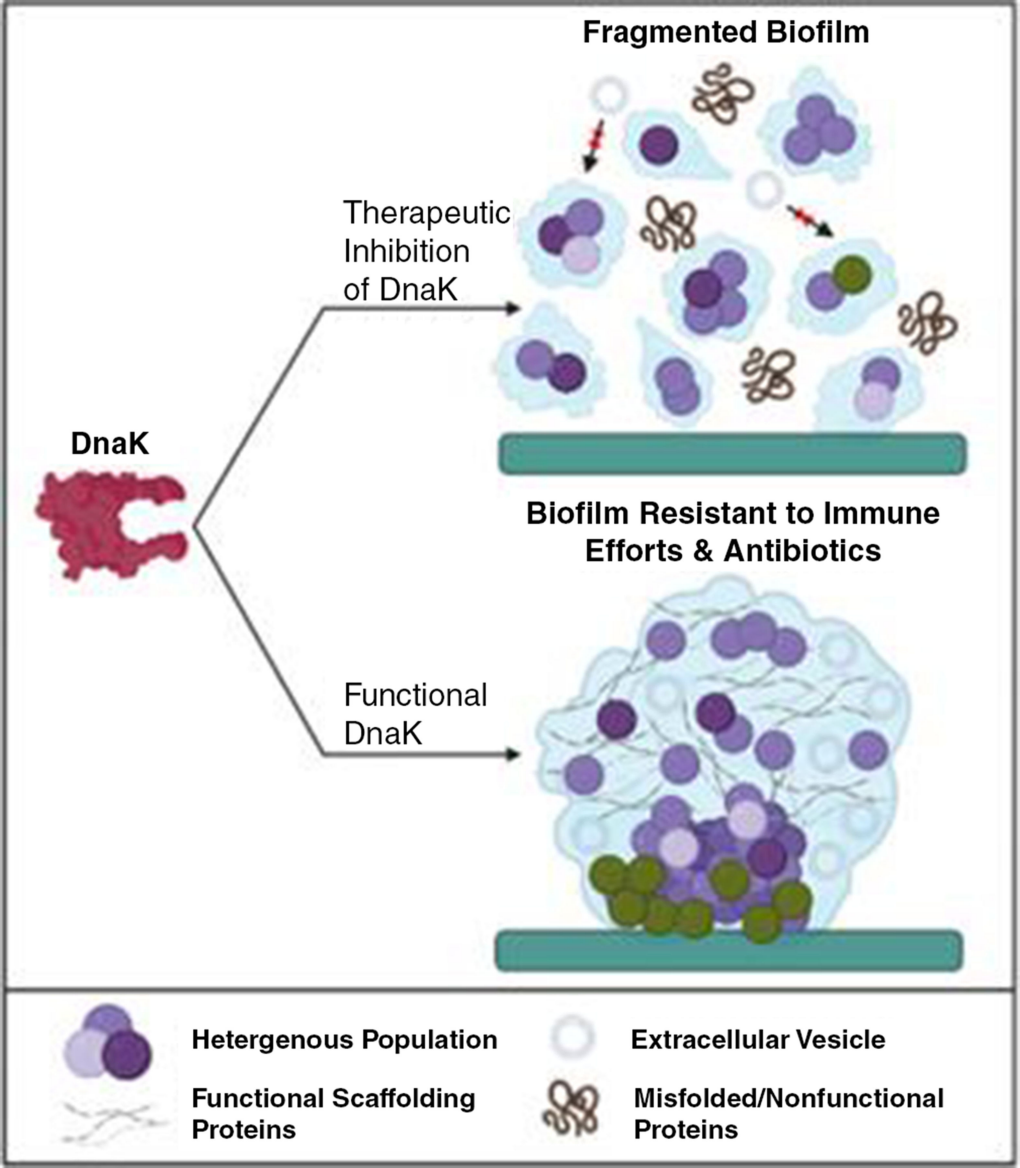
Proposed model for the impact of DnaK inhibition, using SMR peptide, on biofilm structure and intercellular communication. DnaK, a molecular chaperone, is essential to biofilm formation. Although there is a lack of knowledge on the specific mechanism of DnaK, previous studies have identified DnaK as key to amyloid curli expression within the biofilms of *E. coli*. We propose that DnaK is required to appropriately fold functional scaffolding proteins that contribute to the structural integrity of a biofilm. Therefore, we propose that therapeutic inhibition of DnaK with the SMRwt peptide resulted in an accumulation of misfolded proteins with subsequent biofilm degradation. In addition, we theorize that the intercellular communication transit of extracellular vesicles was blocked, by SMRwt targeting of DnaK, further exacerbating dysfunction within the biofilm. Created with BioRender.

In this study, SMRwt peptide was shown to physically interact with DnaK in *E. coli* (Invitrogen MAX Efficiency Stbl2 Competent cells), *S. aureus* (SH1000), and *S. aureus* (SC01) using an immune-precipitation assay with western blotting (Fig. 2A). There was a significant difference between the band percentage intensity of the SMRwt elute compared with the SMRmut elute (Fig. 2B). As SMRwt was shown to interact with DnaK, it likely could interfere with its activity. This would have implications for the regulation of various cellular processes in the bacterium and catalyzed the proposed model for the effect of SMR peptide-mediated DnaK inhibition on biofilm structure and intercellular communication in *S. aureus* (Fig. 5). Given previous studies on the potential of DnaK inhibition, our lab theorized that SMRwt could disrupt *S. aureus* biofilm formation through two primary mechanisms (Fig. 5) as follows: (1) the structural integrity of the biofilm could be compromised through a loss of appropriate folding of scaffolding proteins like amyloid curli. (2) As the SMRwt peptide is capable of EV blockade in both viral infected cells and cancer cells, we postulated that it could similarly affect bacterial intercellular communication networking that is reliant upon EV transmission (52, 53).

In alignment with the proposed DnaK inhibition model, the data illustrated an inhibitory effect of the SMRwt peptide on the biofilm formation of drug-resistant *S. aureus* (SC01) in a dose-dependent manner using the crystal violet staining method and confocal microscopy (Fig. 3 and 4). These inhibitory effects on biofilm formation were not observed with the SMRmut peptide. This supports the expectation that the valine at the first position of the SMRwt peptide is essential for its functionality. Other chemical DnaK inhibitors, such as Myr, have shown a similar inhibitory effect on biofilm formation with a corresponding decrease in amyloid curli (38). SMRwt peptide could serve as a possible anti-bacterial therapeutic by DnaK targeting or as a model for rational drug design to inhibit MDR *S. aureus* biofilm formation (50, 54–70).

In most clinical cases, biofilms are already established (71). Thus, an effective anti-biofilm agent should be capable of disrupting a preformed biofilm (71). We aim to further explore the SMRwt peptide’s impact against preformed biofilms. In preliminary experiments, we observed biofilm disruption following SMRwt-CPP peptide application to a preformed *S. aureus* biofilm. Although the precise mechanism by which SMRwt-CPP disrupts biofilms and the specific bacterial target(s) involved remains unclear, these results provide evidence that this peptide may be a promising anti-biofilm candidate. This research is significant in the broader context of addressing biofilm-related infections, highlighting targets for inhibition, and offering potential solutions with less selective pressure for antibiotic resistance emergence.

### Conclusion

These findings and other data suggest that DnaK antagonism with SMRwt peptide should be investigated further for therapeutic applications. As biofilms act as a platform for promoting antimicrobial resistant tactics, generating anti-biofilm agents will aid in addressing the global threat regarding a dried-up antibiotic discovery pipeline and increasing the prevalence of antibiotic resistance. The SMRwt peptide may be a viable alternative to traditional antibiotics for treating drug-resistant bacteria. However, the exact mechanism of action of the SMRwt peptide is unclear. Determining the mechanism of action for SMRwt peptide biofilm antagonism as well as other clinically relevant parameters, such as *in vivo* efficacy, optimal dosage, and delivery method, will be critical to future development efforts.

## MATERIALS AND METHODS

### Strains and growth conditions

The *S. aureus* SC01 (MRSA) and SH1000 strains were kindly provided by Dr. Jorge Antonio Benitez’s Laboratory and were grown on Mueller–Hinton agar plates with glucose and without antibiotics at 37°C overnight. The *E. coli* (Invitrogen MAX Efficiency Stbl2 Competent cells) strains cells were grown overnight at 30°C in LB Broth without antibiotics.

### Synthesis of SMR-CPPtat and SMR-FLAG peptides

The SMR-CPPtat and SMR-FLAG peptides were custom-made by InnoPep Inc. (San Diego, CA). The amino acid (aa) sequence for the four peptides used in this study is as shown in Fig. 1. SMRwt and SMRmut differ at the first position aa: SMRwt contains a valine, whereas SMRmut contains an alanine. CPPtat is a peptide of arginine and lysine residues that can penetrate the cell membrane. The SMR-FLAG peptides are identical to the SMR-CPP peptides except for the FLAG sequence replacing the CPP sequence.

### Evaluation of biofilm-forming capacity

Strains treated with SMR peptides were screened for their biofilm-forming capacity using MtP methods and a crystal violet assay described by Stepanovic et al. (72). In triplicate, one colony was inoculated to 5 mL of LB or 5 mL of BHI medium and incubated at 37°C overnight with agitation (250 rpm). For evaluating the dose effect of SMR peptides, 20 µL of the overnight cultures to 1 mL LB with 0.5% glucose for *E. coli* or BHI with 2% glucose for *S. aureus* medium with the appropriate concentration of the SMR peptide were mixed well and transferred (100 µL) to 5 wells of the 96 well of the MtP. Negative controls were the SMRmut peptides as well as medium without strains. Plates were incubated at 37°C for *S. aureus* 24 hours without shaking. After the incubation time, the OD at 600 nm was determined using the SpectraMax M5 Microplate Reader. The plates were washed three times with phosphate-buffered saline (PBS) pH 7.4, and adherent bacteria were stained with 0.1% crystal violet. After 30 minutes, the plates were washed three times with PBS, the crystal violet-stained biofilm re-dissolved in dimethylsulfoxide, and the plates read at an OD of 570 nm (OD570). Biofilm production was normalized for growth and expressed as the OD570/OD600 ratio for each well.

### Confocal microscopy

Confocal microscopy was used to visualize biofilm formation. Samples were grown on glass slides and stained with SYTO 9 (Invitrogen, Carlsbad, CA). The biofilms were prepped for confocal microscopy by first growing them in appropriate media for the respective strains with different concentrations of the SMR peptides. After the biofilm formation, the biofilms were stained with 10 µM SYTO9 and incubated at 30°C for 30 minutes in the dark. The biofilms were then washed to remove any extra dye and immediately observed using a TCS-SP2 microscope (Leica Microsystems, Heidelberg, Germany), equipped with a 63 × oil immersion objective. The SYTO9 dye was excited at 483 nm and the fluorescence emission was detected at 503 nm. 3D images were captured every micrometer throughout the biofilm depth. The images were then visualized and processed using the Leica LAS AF software (Leica Microsystems). Quantitative analyses of the images were performed using the COMSTAT software (http://www.imageanalysis.dk/; Heydorn et al., 2000). At least three images were collected from each of the three independent experiments (nine stacks in total) for analysis.

### Immunoprecipitation and Western blot analysis

The interaction between the antagonist and DnaK was determined by immune precipitation with anti-Flag M2 Affinity and Western blot analysis. *E. coli* (Invitrogen MAX Efficiency Stbl2 Competent cells), *S. aureus* (SH1000), and *S. aureus* (SC01) were grown in media without antibiotics and then lysed. The lysate was then screened using anti-flag M2 affinity gel and either the SMRwt or SMRmut peptide, or in the absence of both peptides. The eluted protein samples were separated using SDS-PAGE on 4–20% or 8–16% Tris-HCl Criterion precast gels (Bio-Rad) and transferred to the nitrocellulose membrane, 0.45 µm. The membrane was washed in Tris-Buffered Saline (TBS; Bio-Rad) for 5 minutes, blocked with 5% nonfat milk in TTBS (TBS with 0.1% Tween 20) for 1 hour by shaking at room temperature, processed for immunoblotting using a specific primary mouse DnaK antibody by shaking at 4°C overnight, followed by a secondary HRP-conjugated IgG (H + L) antibody. Protein bands were detected using Western Blotting Luminol Reagent (Santa Cruz Biotechnology, Inc., Santa Cruz, CA) followed by exposure to Image Quant LAS 4000 (FUJIFILM Medical Systems USA, Inc). Densitometry analysis was performed using ImageJ software (National Institutes of Health, Bethesda, MD).

### Statistical analysis

Experiments were performed in three biological replicates with the exception of Figure 3 (n=2). Data obtained from the static biofilm assay were log-transformed to stabilize the variance and to make the approximation to the normal distribution. The statistical analysis was performed using the student’s *t*-test (73–77).
